# Photonic Weyl Waveguide and Saddle-Chips-like Modes

**DOI:** 10.3390/nano14070620

**Published:** 2024-04-01

**Authors:** Hanyu Wang, Wei Xu, Zhihong Zhu, Biao Yang

**Affiliations:** 1 College of Advanced Interdisciplinary Studies, National University of Defense Technology, Changsha 410073, China; wanghanyu0107@163.com (H.W.); weixu08a@163.com (W.X.); 2 Hunan Provincial Key Laboratory of Novel Nano-Optoelectronic Information Materials and Devices, National University of Defense Technology, Changsha 410073, China; 3 Nanhu Laser Laboratory, National University of Defense Technology, Changsha 410073, China

**Keywords:** Weyl meta-crystal, Weyl waveguide, electromagnetic lids, ideal Weyl metamaterial, hybridized waveguide mode, saddle-chips-like modes

## Abstract

Topological Weyl semimetals are characterized by open Fermi arcs on their terminal surfaces, these materials not only changed accepted concepts of the Fermi loop but also enabled many exotic phenomena, such as one-way propagation. The key prerequisite is that the two terminal surfaces have to be well separated, i.e., the Fermi arcs are not allowed to couple with each other. Thus, their interaction was overlooked before. Here, we consider coupled Fermi arcs and propose a Weyl planar waveguide, wherein we found a saddle-chips-like hybridized guiding mode. The hybridized modes consist of three components: surface waves from the top and bottom surfaces and bulk modes inside the Weyl semimetal. The contribution of these three components to the hybridized mode appears to be z-position-dependent rather than uniform. Beyond the conventional waveguide framework, those non-trivial surface states, with their arc-type band structures, exhibit strong selectivity in propagation direction, providing an excellent platform for waveguides. Compared with the conventional waveguide, the propagation direction of hybridized modes exhibits high z-position-dependency. For example, when the probe plane shifts from the top interface to the bottom interface, the component propagating horizontally becomes dimmer, while the component propagating vertically becomes brighter. Experimentally, we drilled periodic holes in metal plates to sandwich an ideal Weyl meta-crystal and characterize the topological guiding mode. Our study shows the intriguing behaviors of topological photonic waveguides, which could lead to beam manipulation, position sensing, and even 3D information processing on photonic chip. The Weyl waveguide also provides a platform for studying the coupling and the interaction between surface and bulk states.

## 1. Introduction

**Figure 1:**
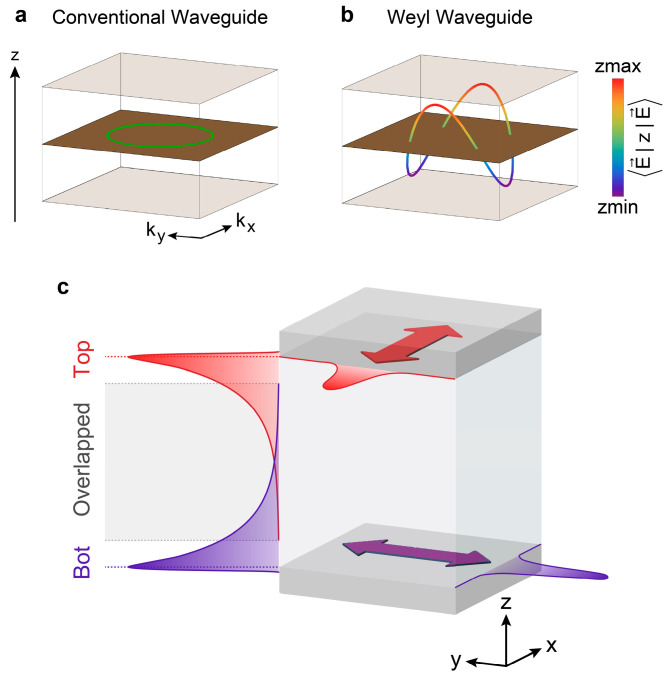
Illustration of propagating modes in conventional and Weyl waveguides at a specific frequency (Weyl frequency). (**a**), The mode in conventional waveguides is distributed uniformly along the z direction. (**b**), Saddle-chips-like propagating mode in a Weyl waveguide, where the propagating direction is locked with the z position. The color bar corresponds to different positions on the z-axis, with red (blue) denoting the top (bottom) boundary of the Weyl waveguide. To clarify the shape of the saddle-chips-like mode, we imagined the central plane of the z-axis of the waveguide and showed it in brown. (**c**), Perspective view of finite-thickness Weyl semimetal with two surfaces. The arrows indicate the in-plane propagation directions of the electromagnetic field. The distribution of the wave function from the top (bottom) interface is colored in red (purple). The dashed red/purple lines denote the position of the top/bottom surface, and the gray dashed lines show the boundary of the overlapping region.

Planar waveguides exhibiting planar geometry guide electromagnetic waves [1,2] in only one dimension. They are often embedded between two substrate layers, such as perfect electric conductors (PEC). Besides their planar geometry, the operation of waveguides mostly relies on the characteristics of the embedded material, which determine the electromagnetic waves traveling in the waveguide in eigenmodes. Considering the projection of the waveguide’s eigenmodes in the z-direction, these modes do not propagate along the waveguide’s top surface at z=h or along the bottom surface at z=0, where h denotes the thickness of waveguide. Instead, they oscillate within the waveguide, between 0≤z≤h. By calculating the centroid of the eigenmode’s projection in the z-direction, it can be observed that the mode centroids are distributed in the middle of the planar waveguide, marked by the green ring in Figure 1a. The propagation of eigenmodes is direction-insensitive in the conventional planar waveguide, which arises from the circle-shaped equi-frequency contour of band structure in the $k_{x}-k_{y}$ plane in the momentum space. The most common method used to manipulate the propagation direction is to change and control the geometric structure of the waveguide, which is challenging in the experimental context. However, we find that the situation is quite different when embedding Weyl meta-crystals, where the eigenmodes are hybridized from both bulk and topological surface modes. This eigenmode in the Weyl waveguide exhibits unique characteristics, including the control and manipulation of the direction of propagation with varying vertical excitation position, as we will discuss in detail later.

Weyl points [3,4,5,6,7,8,9,10,11,12,13] are degenerate points in a three-dimensional gapless system that can exist only when either time-reversal or space-inversion symmetry is broken, or when both are. In momentum space, Weyl points serve as sources and sinks of Berry curvature and can be considered as counterparts of magnetic monopoles. One of the hallmarks of Weyl points is their topologically protected surface states [14,15,16,17], called Fermi arcs. The presence of Fermi arcs is a remarkable property that directly reflects the nontrivial topology of the bulk and plays a key role in the experimental identification of Weyl metamaterials [17,18,19,20,21]. Fermi arcs usually connect the projections of bulk Weyl points [22,23,24,25,26] inside the surface Brillouin zone, in contrast to the conventional closed loop.

It should be noted that Weyl points are defined in the momentum space with three dimensions, that is, infinite size along the three directions x,y,z in real space is required, according to the Fourier transformation. The constraints of periodicity and infinity are easy to meet in theoretical prediction and simulated calculation. However, the infinite size is obviously not feasible and impractical in experimental measurement due to the limitation of sample size. An alternative method involves applying a sample of finite thickness with a large enough the period number; in other words, a sample with a scale much larger than the scale of the wavelength. Thus, the data collected can be approximately considered to be infinite in three-dimensional momentum space. 

Consider the scenario in which the finite thickness of the Weyl semimetal is gradually reduced along the z axis, such that the sample size is no longer much larger than the decay length of the surface state inside the Weyl semimetals. Fermi arcs on the top and bottom surfaces tend to couple with each other and finally form a closed loop, as shown in Figure 1b. The saddle-chip-shaped closed loop bridges the top and bottom surface modes of the Weyl waveguide, exhibiting z-axis-position dependency. In previous studies, the approaches bridging the Fermi arcs of opposite surfaces are from the Weyl orbit [23,27,28,29], i.e., unique momentum-space cyclotron motion. Unlike the typical cyclotron orbits around metallic Fermi surfaces, the propagation of a Weyl orbit through the bulk occurred along chiral Landau levels, where a magnetic field is applied perpendicularly to the surface. Here, we study the coupling between opposite surfaces of a Weyl slab without a magnetic field.

## 2. Materials and Methods

### 2.1. Numerical Simulation

For the bulk band structure (i.e., Weyl nodes), the periodic boundary condition is applied in all three spatial directions. For the Weyl cavity, the PEC boundary condition (add space) is applied to calculate the Fermi arcs in the z direction and the periodic boundary condition is applied along the x and y directions. The Weyl cavity used in numerical simulation was constructed with drilled metallic plates (electromagnetic lids) sandwiching 5 layers of Weyl metacrystals, as is consistent with our experimental setup. The Finite Difference Time Domain (FDTD) method and the Finite Integration Technique (FIT) were applied to calculate the eigenmodes.

### 2.2. Fabrication of the Experimental Sample

The sample was fabricated by a commercial company named Shenzhen Sunsoar Circuit Technology (http://www.oem-pcb.com (accessed on 1 December 2023)) in Shenzhen, China, using the traditional printed circuit board (PCB) fabrication technology. The technological details include: FR4 material, board thickness 3.0 mm, copper weight 1oz, no solder mask and surface leading OSP (organic solder-anility Preservative). For the Weyl meta-crystals, each printed layer was paired with a 1 mm-thick FR4 blank layer to keep the printed copper structures from touching. The probe area consists of 49×49×5 unit cells, whose lattice constants are 7 mm, 7 mm and 4 mm along the x,y and z axes, respectively. An air hole with radius $r_{air}=1.9\;mm$ was drilled at the center of each unit cell, providing the tunnel for the probe antenna to collect fields within the Weyl meta-crystal waveguide. The drilled metallic plates (electromagnetic lids) have a radius r=2.4 mm and a thickness h=3 mm. 

### 2.3. Source and Probe

We employed a microwave vector network analyzer (VNA) and two near-field antennas to act as the source (stationary) and the probe (controlled by an xyz stage). The hole design limits the probe antenna’s scan-step (which determines the maximum range of momentum space $\left[-\frac{\pi }{a},\frac{\pi }{a}\right]$ after Fourier transformation) and z orientation (the probe is mostly sensitive to the field component that is parallel to its orientation). The antenna consists of a coaxial cable with a length of outer conductor and sheath stripped away, leaving the central conductor exposed, which can provide efficient coupling to large-momentum bulk and surface modes. The amplitude and phase of the near field are collected by the antenna with sub-wavelength resolution.

## 3. Results

### 3.1. Topological Waveguide Modes

The surface states of the Weyl semimetal, i.e., Fermi arcs, propagate in-plane, while decay occurs [30,31,32,33] along the out-plane direction, as shown in Figure 1c. The decayed amplitude of the surface state is given by $\vec{E}(k_{x},k_{y})e^{-k_{z}\Delta z}$ at a specific $\left(k_{x},k_{y}\right)$ position, where ∆z represents the distance away from the surface, as shown in Figure 1c. Conventionally, Fermi arcs on the top and bottom surfaces completely decouple, implying that each surface can be treated independently [26,34,35], as long as the thickness of the Weyl semimetals is substantially larger than the decay length. However, when the thickness of Weyl semimetals is gradually reduced, the wave functions of surface states start to overlap and result in hybridization within the Weyl semimetal. The hybridized modes consist of three components: both surface waves from the top and bottom surfaces and bulk modes inside the Weyl semimetal. The contribution of these three components to the hybridized mode appears to be z-position-dependent rather than uniform. For example, at the upper edge of overlapping region, the amplitude of the top Fermi arcs is significantly stronger than that of the bottom Fermi arcs. In the middle position, the weights of the two Fermi arcs are close, while the bulk mode dominates the propagating mode.

**Figure 2:**
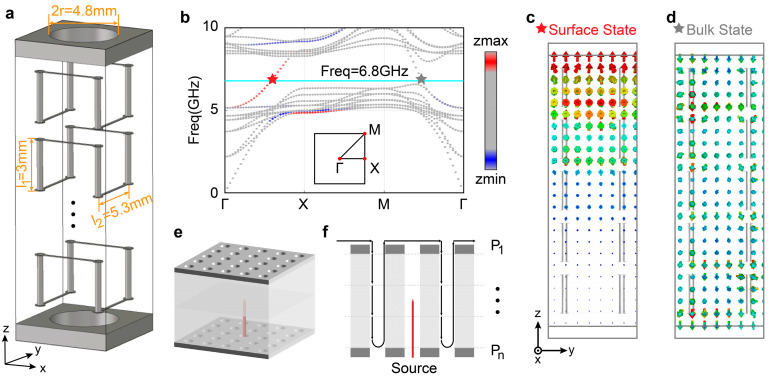
Experimental setups for probing the Weyl planar waveguide. (**a**), Weyl planar waveguide sandwiched between two periodically drilled metallic plates, which enables the raster-scanning z-dependent field distribution. For the drilled metallic plates, the radius of drilled holes is 2.4 mm, and the thickness of the metallic plates is 3 mm. To condense the image, the air holes of the Weyl meta-crystals are not plotted in the figure. (**b**), Band structure along high-symmetry lines. The red (blue) denotes surface states whose wavefunctions are located at the top (bottom) interface. The cyan line denotes the Weyl frequency at 6.8 GHz, and the small square indicates the scan path in the 2D Brillouin zone. The PEC boundary condition is applied along the z direction, and the periodic boundary condition is applied along the x and y directions. (**c**), Mode field of the surface state, marked by a red star in (**b**). The electromagnetic field exhibits a nonuniform distribution along the z-axis, as the field strength is strong near the top interface and decays exponentially as the value of the z-coordinate decreases. (**d**), Mode field of the bulk state, marked by a gray star in (**b**). The electromagnetic field exhibits a uniform distribution within the Weyl meta-crystal. (**e**), The periodic array of (**a**) with an excitation source inserted through the center hole in the bottom surface (red). (**f**), The probe scanning the planar waveguide following up and down cycles (except the central hole). The source antenna is located at the geometric center of the sample.

To realize the hybridized waveguide mode, we sandwiched an ideal Weyl meta-crystal [15] system between two drilled metallic plates, as shown in Figure 2a. The drilled holes are designed to allow the probe antenna to be inserted in the Weyl planar waveguide. The drilled metallic plates behave similarly to PEC boundaries (see the discussion section below), confining the electromagnetic wave inside the Weyl planar waveguide. Following our previous work, see [15], each unit cell of the ideal Weyl meta-crystal consists of a saddle-shaped metallic structure embedded in a dielectric background with a dielectric constant of 2.2. Four Weyl nodes are protected by $D_{2d}$ point group located at the same frequency. Without other non-topological bands around the Weyl nodes, this structure has served as the ideal platform for pursuing various fundamental research and device applications, such as chiral zero modes [36], spiraling phase [14], Veselago lenses [10], etc. To facilitate the subsequent experimental measurement, we further periodically created holes with radius $r_{air}=1.9\;mm$, which preserves the $D_{2d}$ point group and thus also preserves the ideal Weyl nodes. The Weyl meta-crystals and the drilled metallic plates share the same periodicity within the x-y plane, $a_{x}=a_{y}=7\;mm$. In this work, we vertically stacked 5 periods of the ideal Weyl meta-crystal, as shown in Figure 2a. 

The band structure of the Weyl waveguide shown in Figure 2a is given in Figure 2b. As mentioned above, when the thickness of a Weyl semimetal is reduced along the z-direction, the Weyl nodes become ill-defined because the z-extension is too small. As illustrated in Figure 2b, along the high-symmetry line M−Γ, there is only a chiral bulk state intercepted by the cyan line, rather than a Weyl point formed by the intersection of two bands. The band that originally exhibited negative dispersion is removed, leaving only a positive-dispersion bulk band. Furthermore, the finite thickness inevitably leads to non-trivial surface states, that is, Fermi arcs, where surface states on the top and bottom interfaces are denoted in red and blue, respectively, based on the centroid positions of the surface states.

Figure 2e shows the array for experiment setups. The source antenna (red) can be placed at an arbitrary position (x,y,z) to change the excitation position at the Weyl waveguide. Insertion through different holes in the bottom metallic plates allows the source antenna to obtain different in-plane coordinates (x,y), and it can be moved up and down to shift the z position. When measurement begins, the source antenna remains stationary, and the probe antenna moves in the full three-dimensional space to collect data and capture the electromagnetic field distribution in the Weyl waveguide. In Figure 2f, we show the schematic cutting view of the probe scanning path, where the probe antenna raster-scans the interface in a cyclic pattern ($P_{1}\to P_{2}\to P_{n}\to P_{1}\ldots$). The setup allows us to scan all electromagnetic modes inside the Weyl waveguide. The probe antenna skips over the hole occupied by the source antenna during measurement to avoid a collision between the two antennas, as shown in Figure 2f. The influence of the experimental results is negligible because the sample has enough periodicities along the in-plane directions.

### 3.2. Observation of Saddle-Chips-like Mode

**Figure 3:**
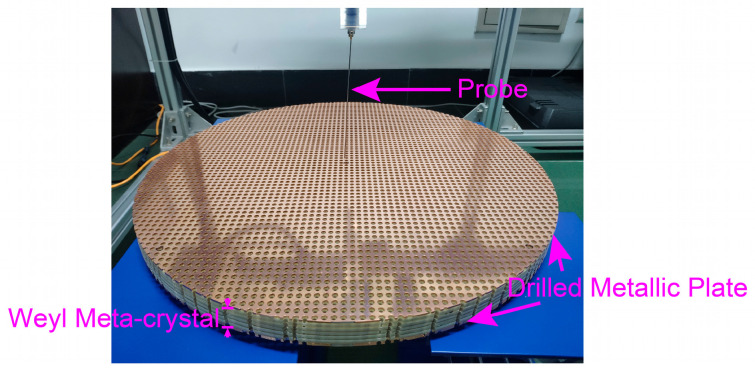
Details of the experimental sample. The 5-layer Weyl meta-crystal is sandwiched between drilled metallic plates with r=2.4 mm. There are 71 unit cells along both the *x* and *y* directions (i.e., its diameter has 71 unit cells). The probe area is a square spanning 49 × 49 unit cells along both the x and y directions (see details in Methods).

**Figure 4:**
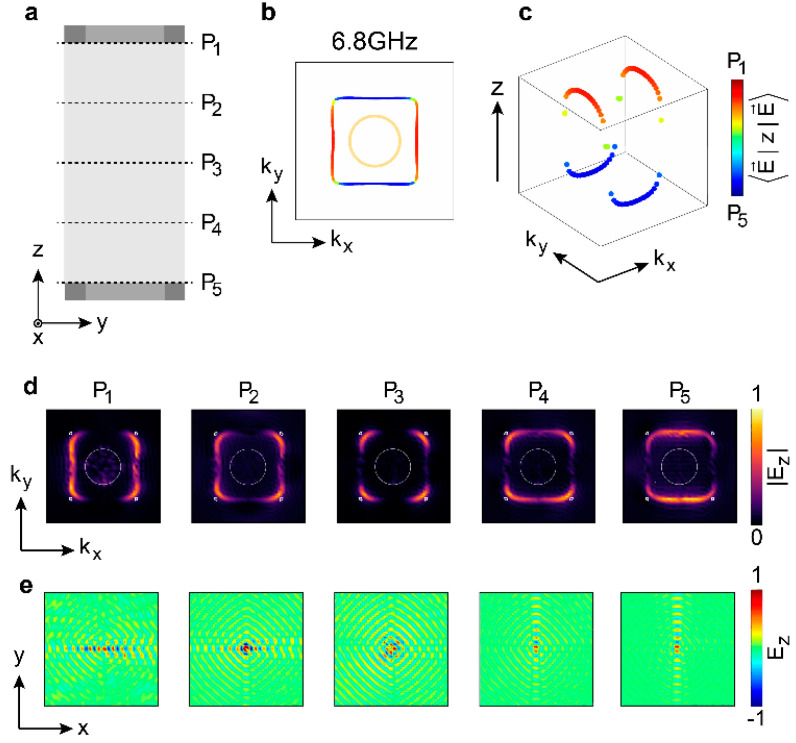
Experimentally mapped saddle-chips-like mode in the Weyl planar waveguide at 6.8 GHz. (**a**), Five cuts along the z direction are swept within the waveguide. (**b**), Simulated projected hybridized waveguide modes in the first Brillouin zone. The yellow circle denotes the light cone. (**c**), Perspective view of (**b**), illustration of the z-position-dependent equifrequency contour, which forms the saddle-chips-like mode. (**d**), Experimentally mapped energy distribution at the corresponding positions. The light cone is denoted by the white circle. The positions of projected Weyl points are also marked by white dots to compare them with the waveguide modes, even though Weyl points are ill-defined in the experiment. The source antenna is placed at $P_{2}(P_{4})$ in the central hole to excite the Fermi arcs of the top (bottom) surface. That is, the pattern of $P_{1}$ and $P_{2}$ is probed when the source is placed at $P_{2}$, and $P_{3}-P_{5}$ is probed when the source is placed at $P_{4}$. (**e**), The propagation direction turns 90 degrees when the z position is swept in real space, as shown in (**d**). The probe area consists of 49 × 49 × 5 unit cells, whose lattice constants are 7 mm, 7 mm and 4 mm along *x*, *y* and *z* axes, respectively.

The experimental sample is illustrated in Figure 3. The microwave near-field scanning system is applied to probe the electromagnetic field, which is composed of a microwave vector network analyzer (VNA), two near-field antennas, and an xyz stage. To map the evolution of the propagating direction of the waveguide mode, we experimentally scanned 5 distinct positions within the ideal Weyl waveguide, which were referred to as $P_{1}$ to $P_{5}$, as shown in Figure 4a. The waveguide mode at $P_{1}(P_{5})$ propagated along $k_{x}(k_{y})$ as shown in Figure 4b,c; the experimental probed band structure is shown in Figure 4d, with its real space field distribution in Figure 4e. Here, $\left|\vec{E}\right\rangle$ denotes the electric field, and $\left\langle \vec{E}\left|z\right|\vec{E}\right\rangle$ is the weighted position of the mode distribution in the z direction and is shown by a different marking color.

Figure 4b,c shows the simulated projected hybridized waveguide modes in the first Brillouin zone at 6.8 GHz. Based on the centroid position of each point, we further applied colors, with red (blue) representing centroids located at the top (bottom) interface. To demonstrate the centroid positions of the modes more clearly, we introduced the z-axis in Figure 4c. The hybridization within the Weyl waveguide exhibits pronounced position dependence along the z-axis (see Figure 4c). For an ideal Weyl semimetal with a large enough z-extension that the top and bottom surfaces are completely decoupled, the mode centroid distribution along the z axis is divided into three parts: top-surface Fermi arcs (red), bottom-surface Fermi arcs (blue), and the Weyl bulk states with centroid positions at the sample center (green). These three parts are fully decoupled, and the mode distribution along the z axis is discrete. In the Weyl waveguide, around the position nearing the Weyl cones, the Fermi arcs from the top (bottom) surface move along $z_{min}(z_{max})$, heading toward the sample center. Hybridization changes the distribution of Fermi arcs along the z direction and combines the top and bottom surface states with the bulk states to form a smooth saddle-chips-like mode, as shown in Figure 4c.

Figure 4d shows the band structure in momentum-space at 6.8 GHz. Collecting the field distributions for different planes $\left(P_{1}-P_{5}\right)$ allows the real-space pattern in Figure 4e to be obtained. Applying the Fourier transformation of the real-space pattern yields the band structure in momentum-space. The range of momentum space is $\left[-\frac{\pi }{a},\frac{\pi }{a}\right]$, as the hole design limits the probe antenna’s scan-step. At the bottom surface $P_{5}$, the waveguide modes rotate 90° compared with $P_{1}$, as illustrated in Figure 4d, which is consistent with the simulation results. As these two sets of waveguide modes (i.e., at $P_{1}$ and $P_{5}$) are orthogonal to each other, the middle position exhibits intriguing interference patterns. In particular, at $P_{3}$, the coupling bends the hybridized modes and the centroid from the end of the top (bottom) Fermi arcs shifts along z axis, toward the sample center, forming four rounded corners (see Figure 4d), as mentioned before. In an overall view, moving the probe plane inside the Weyl cavity from $P_{1}$ to $P_{5}$, the waveguide mode from the top surface decreases gradually, while that from the bottom surface increases. The real-space electromagnetic field pattern in Figure 4e provides another perspective to aid in understanding the saddle-chips-like mode. The propagation direction of hybridized modes correspondingly exhibits high z-position-dependency, i.e., the component propagating horizontally becoming dimmer, while the component propagating vertically becomes brighter.

## 4. Discussion

Above, we discussed the Weyl waveguide modes between two drilled metallic plates. Here, we argue that the drilled metallic plate plays a role similar to that of a PEC. PECs are ideal trivial backgrounds for the study of topological photonics. However, PEC boundaries pose a major challenge for experimental characterization, i.e., the probe cannot directly detect the surface states, while a probing antenna inserted from the side will inevitably introduce an unnecessary air gap. Thus, the direct experimental detection of Fermi arcs on a PEC boundary remains elusive.

**Figure 5:**
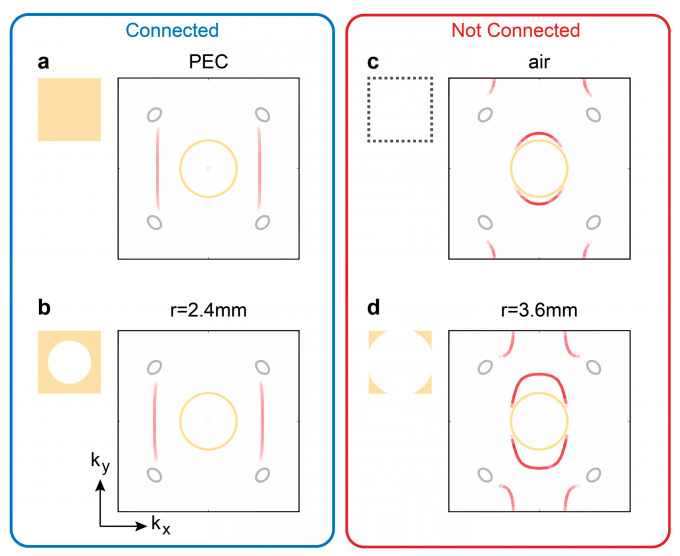
Drilled metallic plates behave like the perfect electric conductor (PEC) boundary. (**a**,**b**), When the radius r<a/2, the drilled metallic plates behave like PEC boundaries. (**c**,**d**), When the radius r>a/2, the drilled metallic plates behave like open boundaries (i.e., air). The frequency here (6.7 GHz) is lower than the Weyl frequency (6.8 GHz), so the Weyl point exhibits a larger projected cross-section. When the metallic structures between unit cells are not connected (r>a/2), there exist trivial surface states akin to the light cone.

To address this difficulty, we proposed using the drilled metallic plates. Given that the Weyl point frequency is around 6.8 GHz, corresponding to a wavelength of approximately 44 mm—significantly larger than the aperture radius r—this drilled metallic plate structure retains robust confinement of electromagnetic waves. Figure 5a gives the top surface states with the PEC boundary. When the drilled radius r=2.4 mm, the morphology of the Fermi arcs, as shown in Figure 5b, closely resembles that of the PEC in Figure 5a. This finding suggests that the drilled metallic plate behaves similarly to PEC in that it provides good confinement of the electromagnetic fields, leading us to dub it an “electromagnetic lid”. When the radius of the drilled hole is large enough, i.e., r>a/2, the drilled metallic plates approach the air boundary, as shown in Figure 5c,d.

## 5. Conclusions

In this article, we introduced a Weyl waveguide composed of an ideal Weyl meta-crystal sandwiched between two drilled metallic plates. This waveguide supports a guiding mode propagating along perpendicular directions within the x-y plane when vertically shifting excitation positions along the z-axis. Given its highly directional field propagation, it may find potential applications in the design of filters and beam splitters, among other conventional electromagnetic devices. The z-axis position dependency provides opportunities for use in sensing applications. In the realm of information transmission, its inherent dual-channel capabilities along the x-axis and y-axis, where the channels are orthogonal, endow it with robust interference resistance. By adjusting the position of the ideal Weyl cavity, one can selectively activate individual channels or concurrently engage both channels, paving the way for next-generation multiplexers or parallel computing architectures.

In the Weyl waveguide, similarly to what is seen in conventional waveguides, bulk states (Weyl cones) are involved in the guiding of electromagnetic waves. Beyond the conventional waveguide framework, those non-trivial surface states, with their arc-type band structures, exhibit strong selectivity in terms of the propagation direction, providing an excellent platform for waveguides based on non-trivial surface states. Specifically, due to the unique physical properties of Fermi arcs, it can be anticipated that Fermi arcs will offer a wealth of phenomena for the Weyl waveguide, such as circumventing obstructions.

The Weyl waveguide also provides a platform for discussing the coupling and interaction between surface states and bulk states. In previous work, the coupling between surface and bulk states, or between different surface states, was typically solved using tight-binding models, directly assigning values to coupling coefficients. The Weyl waveguide offers an excellent system for surface-bulk-surface state coupling, where adjusting the number of layers in Weyl meta-crystals can achieve the purpose of modulating the coupling strength in actual structures. Furthermore, by changing the boundary conditions of the Weyl waveguide, such as by switching from PEC to air or PMC, the interactions among various surface states can be discussed.

Note added.—After finishing this manuscript, we became aware of a related eprint by Han et al. [37], in which a family of topological chiral bulk states extends over photonic Weyl meta-crystal waveguides.

## Data Availability

Data underlying the results presented in this paper are not publicly available at this time but may be obtained from the authors upon reasonable request.
